# Upregulated Transcription Factor PITX1 Predicts Poor Prognosis in Kidney Renal Clear Cell Carcinoma-Based Bioinformatic Analysis and Experimental Verification

**DOI:** 10.1155/2021/7694239

**Published:** 2021-11-23

**Authors:** Yinglang Zhang, Zhe Zhang, Wei Zhang, Hailong Hu, Guochang Bao

**Affiliations:** ^1^Department of Urology, Affiliated Hospital of Chifeng University, 024000 Chifeng, Inner Mongolia, China; ^2^Department of Urology, The Second Hospital of Tianjin Medical University, 300211 Tianjin, China

## Abstract

Paired-like homeodomain transcription factor 1 (PITX1) is involved in numerous biological processes, including cell growth, progression, and invasion in various malignant tumors. Nevertheless, the relationship between PITX1 and kidney renal clear cell carcinoma (KIRC) remains unclear. The clinical role and functions of PITX1 were analyzed by integrating multiple open-access online datasets. Further experimental verification was performed via quantitative real-time PCR (qRT-PCR) to detect the expression of PITX1 in 10 pairs of KIRC tissues. Our results revealed that PITX1 mRNA was overexpressed in tumor tissues compared with normal tissues in the TCGA-KIRC database (*p* < 0.001) and numerous independent cohorts (*p* < 0.05). Further, high expression of PITX1 mRNA was detected in KIRC tissues compared with adjacent normal tissues in our center by qRT-PCR (*N* = 10, *p* < 0.05). Logistic regression analysis demonstrated that the PITX1 level was positively associated with KIRC patients, T and M stages, histologic grade, and pathologic stage (all *p* < 0.05). Survival analysis showed that upregulation of PITX1 mRNA was associated with poor overall survival (OS), disease-free survival (DFS), and disease-specific survival (DSS) (all *p* < 0.05). Univariate/multivariate Cox hazard regression analysis revealed that PITX1 was an independent risk factor for OS in patients with KIRC (HR = 1.998, *p* = 0.003). Accordingly, the time-independent receiver operating characteristic (ROC) curve confirmed that PITX1 had good predictive efficacy for OS and DSS. Meanwhile, a prediction model constructed by nomogram was used to predict the OS of KIRC patients, and the calibration plot indicated this model shows high accuracy. We also revealed some downstream target genes of PITX1-related signaling pathways. Our finding suggested that high PITX1 mRNA expression may act as an independent predictive factor of poor prognosis in patients with KIRC. The prognostic model based on the nomogram would be instrumental in evaluating the survival rate in KIRC patients.

## 1. Introduction

Renal cell carcinoma (RCC) is one of the most lethal urological malignancies. Currently, novel cases of RCC have skyrocketed worldwide with approximately 400,000 cases emerging each year [1]. Kidney renal clear cell carcinoma (KIRC) is considered the most aggressive histological type, accounting for 70–80% of all RCC cases [2]. Following curative treatment for localized KIRC, up to 30% of patients may experience tumor recurrence or metastasis after being considered disease-free, leading to an unsatisfactory prognosis [3]. The current conventional prognostic assessment for KIRC mainly depends on the clinical TNM stage and pathological staging. Tumor heterogeneity and complex pathogenesis often affect the predictive efficacy for the overall survival of patients with KIRC. Therefore, there is an urgent need to develop sensitive and reliable prognostic models to complement the predictive outcomes of clinical pathological features and to optimize the management of KIRC patients.

Genome-sequencing technology has revealed the prognostic potency of gene signatures for KIRC. Publicly available genomic data can be used to identify multiple and efficient prognostic gene signatures. These potential molecular biomarkers for KIRC prognosis include mRNAs, circRNAs, lncRNAs, and miRNAs [4–7]. PITX1 is located on a chromosome 5q31 and is a member of the bicoid-related homeobox transcription factor (TF) gene family that participates in the transcription of the proopiomelanocortin (POMC) gene [8]. Numerous studies have confirmed that PITX1 plays a vital role in proper development, such as in the pituitary gland, craniofacial structures, hind limbs in early embryonic development, and adolescent idiopathic scoliosis [9–11]. Differential expression of *PITX1* has been reported in many malignant tumors and is associated with the survival prognosis of patients in several types of cancers, such as lung adenocarcinoma [12], osteosarcoma [8], esophageal squamous carcinoma [13], and head and neck squamous cell carcinoma [14].

To date, the prognostic role of PITX1 mRNA in KIRC remains elusive after retrieving in PubMed (https://pubmed.ncbi.nlm.nih.gov). In this investigation, our aim was to determine the correlation between PITX1 mRNA expression and the clinical prognosis of KIRC by using TCGA-KIRC expression profiles and various bioinformatic online databases, thus clarifying the prognostic significance of the PITX1 gene in KIRC patients.

## 2. Materials and Methods

### 2.1. Clinical Tissue Specimens

The 10 pairs of KIRC and matched adjacent normal tissues were obtained at the Second Hospital of Tianjin Medical University during May 2021. All patients had undergone robot-assisted or laparoscopic radical resection without any preoperative chemotherapy, radiotherapy, and immunotherapy. These fresh tissues were immediately frozen in liquid nitrogen after surgical removal and stored at −80°C until RNA extraction. The present study was approved by the Ethics Committee of the Second Hospital of Tianjin Medical University. Each patient signed written informed consent forms. Basic information and imaging data of these patients are shown in Table S1 and Figure S1.

### 2.2. RNA Preparation and qRT-PCR

Total RNA was extracted from tissue samples using the HP Total RNA Kit (Omega Bio-Tek, USA) and reverse transcribed into cDNA with RevertAid First Strand cDNA Synthesis Kit (Thermo Fisher Scientific, USA). qRT-PCR was performed on a Quantagene q225 system (Kubo Tech, China) using the TOROGreen qPCR Master Mix (Toroivd, Shanghai); GAPDH was used as an internal reference and the 2^-△△CT^ method was used to calculate the relative expression of PITX1. Primers were synthesized by Sangon Biotech (Shanghai, China). The sequence of GAPDH was forward primer 5′-CGGAGTCAACGGATTTGGTC-3′ and reverse primer 5′-TTCCCGTTCTCAGCCTTGAC-3′, and the sequence of PITX1 was forward primer 5′-GACCCAGCCAAGAAGAAGAA-3′ and reverse primer 5′-AACTGCTGGCTTGTGAAGTG-3′.

### 2.3. Data Acquisition and Differential Expression Analysis

PITX1 mRNA expression was analyzed in pancancer based on the TCGA database using the Tumor Immune Estimation Resource website (TIMER, https://cistrome.shinyapps.io/timer/). Gene mRNA expression profiles and clinical data of KIRC patients were downloaded from The Cancer Genome Atlas (TCGA, https://portal.gdc.cancer.gov) database. TCGA-KIRC-RNA-seq-TPM dataset contained data for 539 tumor samples and 72 normal kidney tissue samples, each containing the following patient details: age, sex, race, tumor laterality, TNM stage, histologic grade, pathologic stage, primary therapy outcome, and prognostic information. To further verify the expression of PITX1 mRNA in KIRC tissues, four PITX1 mRNA expression profiles (Yusenko, Lenburg, GSE53757, and GSE16441 renal clear cell cancer cohorts) were obtained from the Oncomine (https://www.oncomine.org) database and the Gene Expression Omnibus (GEO, https://www.ncbi.nlm.nih.gov) database. For public data, we used 3.6.3 R package to perform all statistical analyses and visualization, the expression of PITX1 mRNA between KIRC and unpaired tissues by Wilcoxon rank sum test, and paired tissues by Wilcoxon signed rank test. The associations of PITX1 mRNA expression with clinical features were analyzed using the Wilcoxon test or Kruskal–Wallis test and logistic regression. For our clinical samples, paired *t* test method and GraphPad Prism 9 software were applied to perform statistical analysis. Differences were defined as significant at a *p* value < 0.05.

### 2.4. Survival Analyses

High and low PITX1 mRNA expression groups were cut-off values by median values. Survival analyses in the TCGA-KIRC cohort were conducted through Kaplan–Meier (KM) survival curve analysis with log-rank test. OS and DFS were analyzed using the online web databases OncoLnc (http://www.oncolnc.org) and Gene Expression Profiling Interactive Analysis (GEPIA, http://gepia.cancer-pku.cn). DSS and OS of subgroups were statistically analyzed using the survival R package and visualized with the survminer R package. Time-dependent receiver operating characteristic (ROC) curve analyses were performed to assess the predictive capacity of PITX1 in OS and DSS. timeROC package and ggplot2 R package were used for analysis and visualization, respectively.

### 2.5. Independent Prognostic Value of PITX1 mRNA

To identify the independent prognostic value of PITX1 in KIRC and assess the correlation between crucial clinical characteristics and prognosis, we performed Cox analyses. First, we conducted a univariate Cox analysis on each variable to determine its correlation with prognosis. Next, we conducted a multivariate Cox analysis on all variables to evaluate whether each of them was an independent prognostic factor. OS was selected as the dependent variable to calculate the hazard ratio (HR) and the 95% confidence interval (two-sided *p* value). From the results of these analyses, a nomogram model, which is a tool commonly used to predict certain clinical outcomes or the probability of an event occurring, was constructed to predict the OS of KIRC patients, and calibration curves were used to evaluate the accuracy of the model using the rms R package and survival R package.

### 2.6. Genes Coexpressed with PITX1 and Protein-Protein (PPI) Interaction Analysis

We used the 3.6.3 R package to analyze the differentially expressed genes related to PITX1 from the TCGA-KIRC cohort. The Spearman test was used to perform statistical analysis, and the correlation of results was presented in heat maps. The GEPIA2 (http://gepia2.cancer-pku.cn/#index) database was used to plot survival heat maps of the top coexpression genes. STRING (Version 11.0, http://string-db.org), a database used to consolidate known and predicted protein-protein association data, was used to set up a protein-protein interaction (PPI) network, and a visualized diagram was acquired using Cytoscape (version 3.7.1).

### 2.7. Biological Function Analysis of PITX1

PITX1 acts as a TF; the Transcriptional Regulatory Relationships Unraveled by Sentence-based Text mining (TRRUST version 2,https://www.grnpedia.org/trrust/), which contains 8,444 TF-target regulatory relationships of 800 human TFs, was used to analyze the relationship between PITX1 and its target genes. Accordingly, the Gene Ontology biological process (GO-BP) and Kyoto Encyclopedia of Genes and Genomes (KEGG) pathway PITX1 enriched were analyzed, as well. Statistical significance was set at *p* < 0.05 and false discovery rate (FDR) *q* < 0.25.

## 3. Results

### 3.1. Clinicopathologic Features of Patients with KIRC

A total of 539 patients from the TCGA-KIRC cohort who presented with the required clinical characteristics were included. A detailed list is provided in Table 1. Among them, 270 (50.1%) were over 60 years old. 186 (34.5%) were female, and 353 (65.5%) were male. 467 (87.8%) patients were white race, which took up the majority. 252 (46%) were found to have tumors on the left side and 286 (53.2%) on the right side. T1 stage was found in 278 (51.6%), T2 in 71 (13.2%), T3 in 179 (33.2%), and T4 in 11 (2%). However, only 16 (6.2%) and 78 (15.4%) patients had lymph node metastasis and distant metastases, respectively. 272 (50.7%) were in pathologic stage I, 59 (11%) were in pathologic stage II, 123 (22.9%) were in pathologic stage I, 82 (15.3%) were in pathologic stage IV. As for primary therapy outcome, 128 (87.1%) reached CR. Besides, 366 (67.9%) were alive in the OS event. 420 (79.5%) were alive in the DSS event. 378 (70.1%) were alive in the PFI event.

### 3.2. High PITX1 mRNA Expression in KIRC

**Figure 1:**
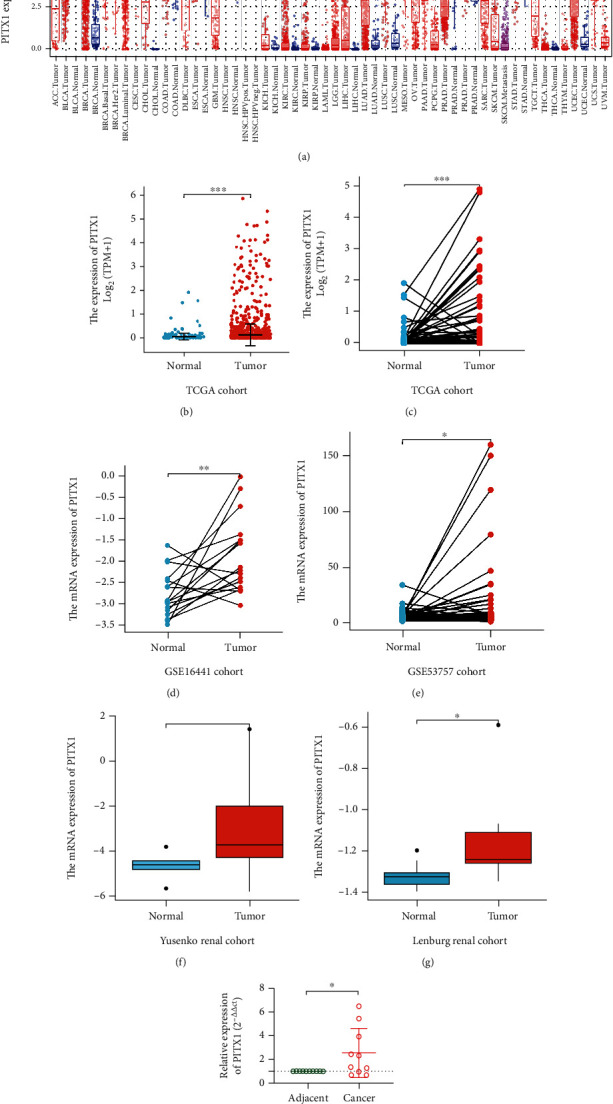
PITX1 is highly expressed in KIRC. (a) PITX1 expression in pancancers. (b) Unpaired tumor and normal tissues from the TCGA-KIRC dataset (T = 539, N = 72). (c) Paired tissues from the TCGA-KIRC dataset (T = 72, N = 72). (d) Paired tissues from the GSE16441-KIRC dataset (T = 17, N = 17). (e) Paired tissues from the GSE53757-KIRC (T = 72, N = 72). (f) PITX1mRNA expression in the Yusenko renal cohort (T = 26, N = 5). (g) PITX1 mRNA expression in the Lenburg renal cohort (T = 9, N = 9). (h) Relative expression of PITX1 mRNA in KIRC tissues and adjacent nontumor tissues was detected by qRT-PCR (N = 10).

PITX1 mRNA expression was summarized in pancancers from the TGCA database (Figure 1(a)). It was easy to find that PITX1 mRNA expression was different in various types of cancers. However, both unpaired and paired tests indicated that PITX1 mRNA expression was regulated in KIRC tissues (*p* < 0.05) (Figures 1(b)–1(g)). We also detected the expression of PITX1via qRT-PCR in 10 pairs of tumor samples and paracarcinoma samples from KIRC patients in our center. The result showed that the PITX1 mRNA level was higher in KIRC tissues than in matching adjacent normal tissues (*p* < 0.05) (Figure 1(h)), which is in line with the analysis of these databases.

### 3.3. Relationships between PITX1 mRNA Expression and Clinical Characteristics

**Figure 2:**
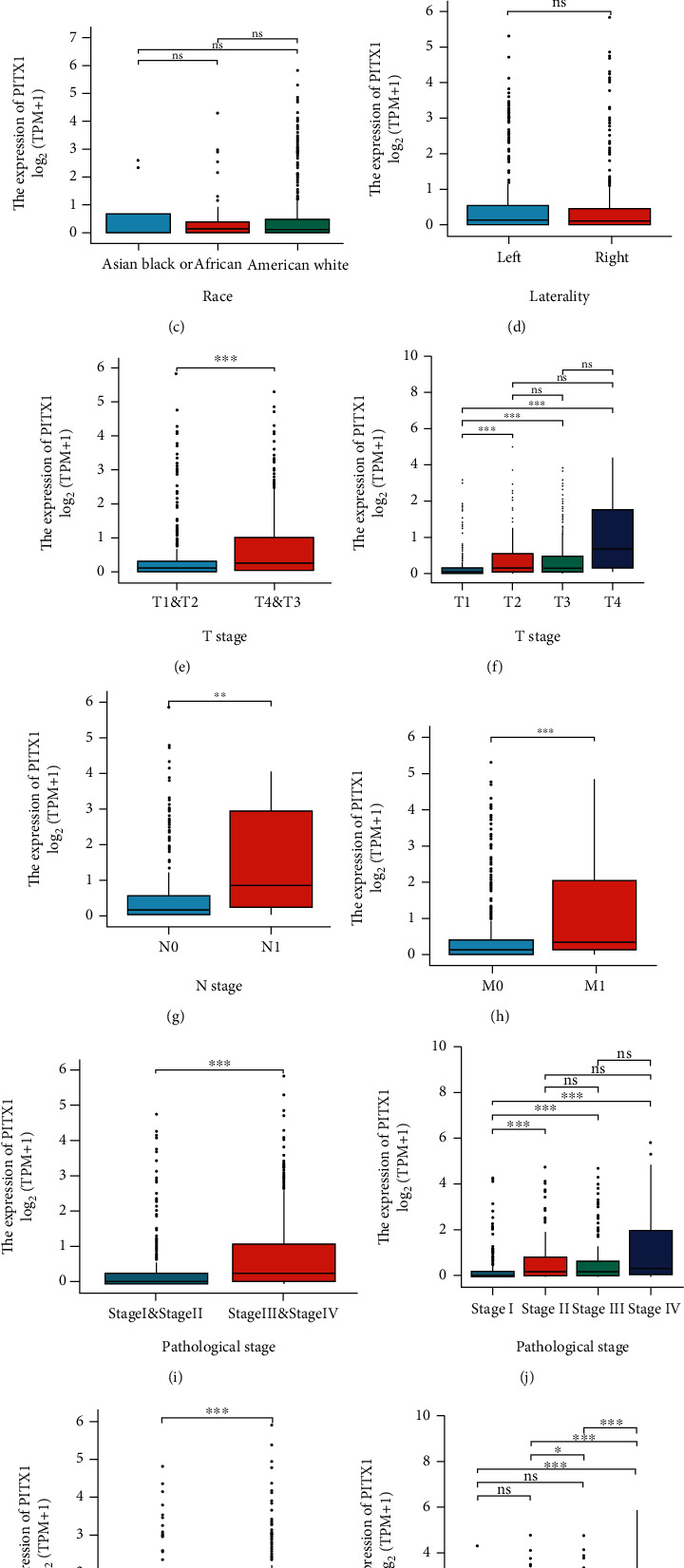
The relationship between PITX1 mRNA expression level and clinical characteristics: (a) age; (b) gender; (c) race; (d) laterality of tumor; (e, f) T stage; (g) N stage; (h) M stage; (i, j) pathologic stage; (k, l) histologic grade.

As shown in Figure 2, high expression of PITX1 was significantly associated with age (*p* < 0.05), T stage (*p* < 0.001), N stage (*p* < 0.01), M stage (*p* < 0.001), histologic stage (*p* < 0.001), and pathologic stage (*p* < 0.001). However, no statistical differences were found in gender, race, and tumor laterality (*p* > 0.05). Logistic regression was used to assess the relationship between PITX1 expression and clinicopathologic features (Table 2). There existed significantly associations between high PITX1 expression and T stage (OR = 2.354, T3+T4 vs. T1+T2, *p* < 0.001), M stage (OR = 2.939, M1 vs. M0, *p* < 0.001), pathologic stage (OR = 2.775, stage III+IV vs. stage I+II, *p* < 0.001), and histologic grade (OR = 2.384, G3+G4 vs. G1+G2, *p* < 0.001). Briefly, a close correlation was detected between evaluated PITX1 mRNA and worse clinicopathologic parameters.

### 3.4. PITX1 mRNA Could Be an Independent Prognostic Factor for KIRC

**Figure 3:**
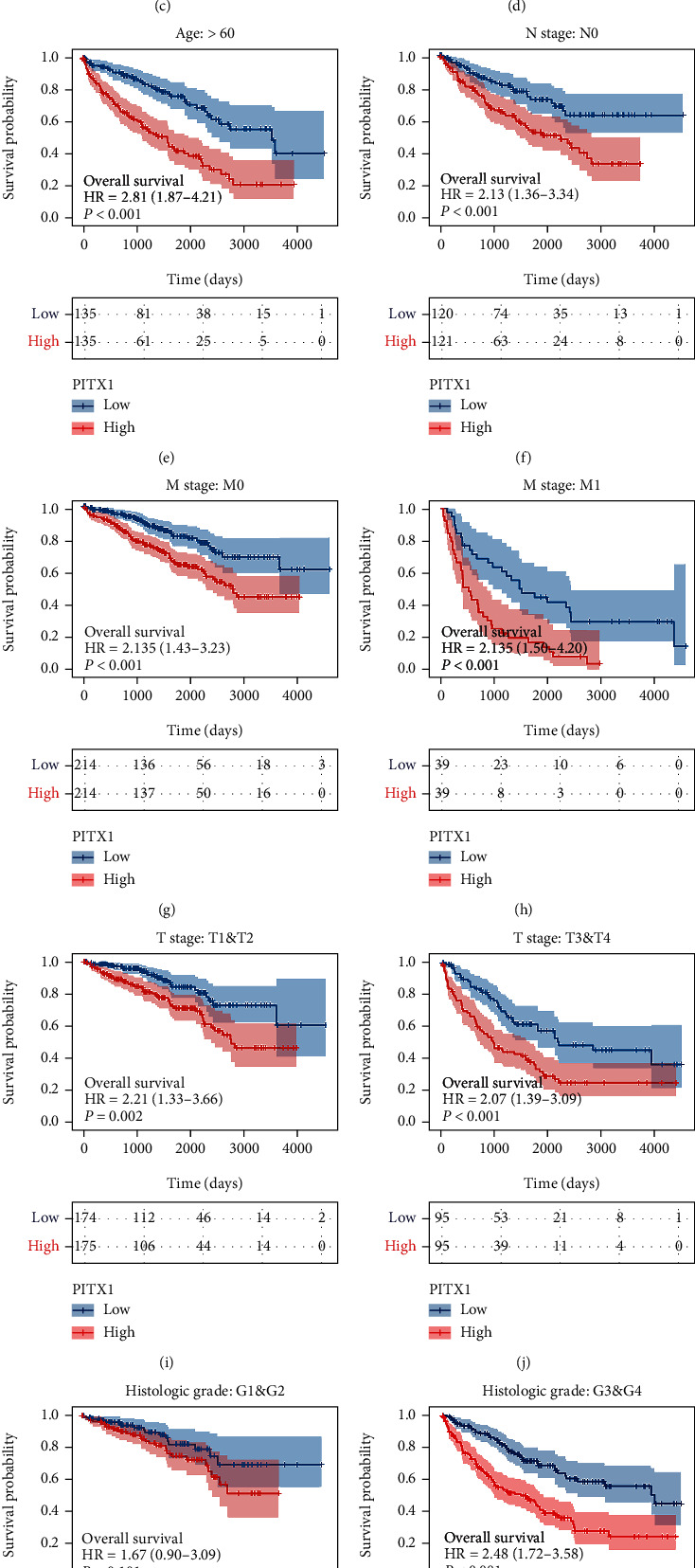
Kaplan-Meier curve for OS, DFS, and DSS in KIRC. (a, b) Kaplan-Meier survival analyses for OS and DFS of PITX1 mRNA expression stemmed from OncoLnc and GEPIA online databases, respectively. (c) Kaplan-Meier survival analysis for DSS of PITX1 mRNA expression. (d–l) Subgroup analysis for ages ≤ 60/>60, N0, M0, M1, stage T1/T2, stage T3/T4, histologic G1/G2, and histologic G3/G4. (m, n) Time-independent ROC curves for OS and DSS. OS: overall survival; DFS: disease-free survival; DSS: disease-specific survival.

**Figure 4:**
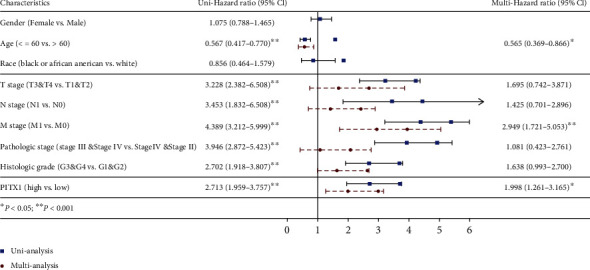
Forest plot of the univariate and multivariate Cox regression analyses in KIRC.

**Figure 5:**
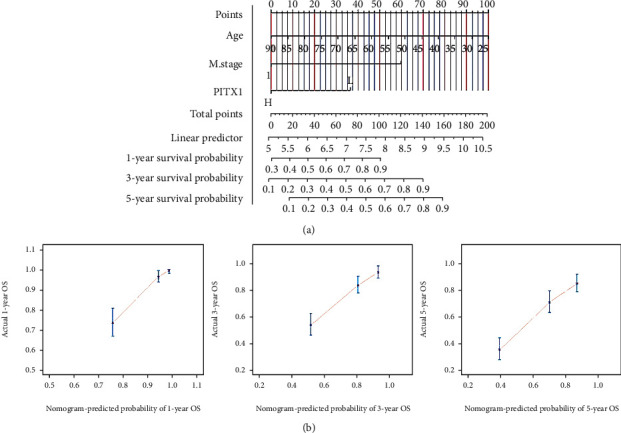
Nomogram for overall survival prediction. (a) A nomogram combining with clinicopathological features and PITX1 mRNA expression. (b) Calibration plot shows that predicted survival probabilities by nomogram align closely with the actual proportions of incidence. *X*-axis is nomogram-predicted probability of OS; *Y*-axis is observed OS. OS: overall survival.

Kaplan-Meier survival analysis demonstrated that high PITX1 mRNA expression was associated with poor OS (*p* < 0.001), DFS (*p* < 0.001), and DSS (*p* < 0.001) (Figures 3(a)–3(c)). Subgroup analysis by different clinical features showed that high expression of PITX1 was significantly associated with poor OS prognosis in KIRC cases ages ≤60/>60 years (*p* < 0.001), N0 (*p* = 0.001), M0 (*p* < 0.001), M1 (*p* < 0.001), stage T1/T2 (*p* = 0.002), stage T3/T4 (*p* < 0.001), and histologic G3/G4 (*p* < 0.001) (Figures 3(d)–3(j) and 3(l)). Time-dependent ROC curves with OS and DSS as endpoints were generated, and a higher area under the curve (AUC) indicated better the model performance. The AUCs of the PITX1 signature model corresponding to 1, 2, and 3 years of OS and DSS are shown in Figures 3(m) and 3(n). To confirm whether PITX1 can be used as an independent prognostic indicator, we performed univariate and multivariate Cox regression analyses. Univariate Cox analysis suggested that age, TNM stage, pathologic stage, histologic grade, and PITX1 expression were related to OS (all *p* < 0.001). Multivariate Cox analysis showed that only age (≤60 vs. >60, HR = 0.565, 95% CI: 0.369-0.866, *p* = 0.009), M stage (M1 vs. M0, HR = 2.949, 95% CI: 1.721-5.053, *p* < 0.001), and PITX1 expression level (high vs. low, HR = 1.998, 95% CI: 1.261-3.165, *p* = 0.003) were associated with OS (*p* < 0.05) (Table 3 and Figure 4). Subsequently, a nomogram was constructed to predict the 1-, 3-, and 5-year survival probability of patients by combining the expression of PITX1 mRNA and clinical variables; the c-index of the nomogram was 0.739 (95% CI: 0.720-0.758) (Figure 5(a)). By calibration curve analysis, we found that the survival probabilities predicted by the nomogram were extremely close to the observed survival probability, which further confirmed the reliability of the nomogram (Figure 5(b)).

### 3.5. PITX1 Coexpression Network in KIRC

**Figure 6:**
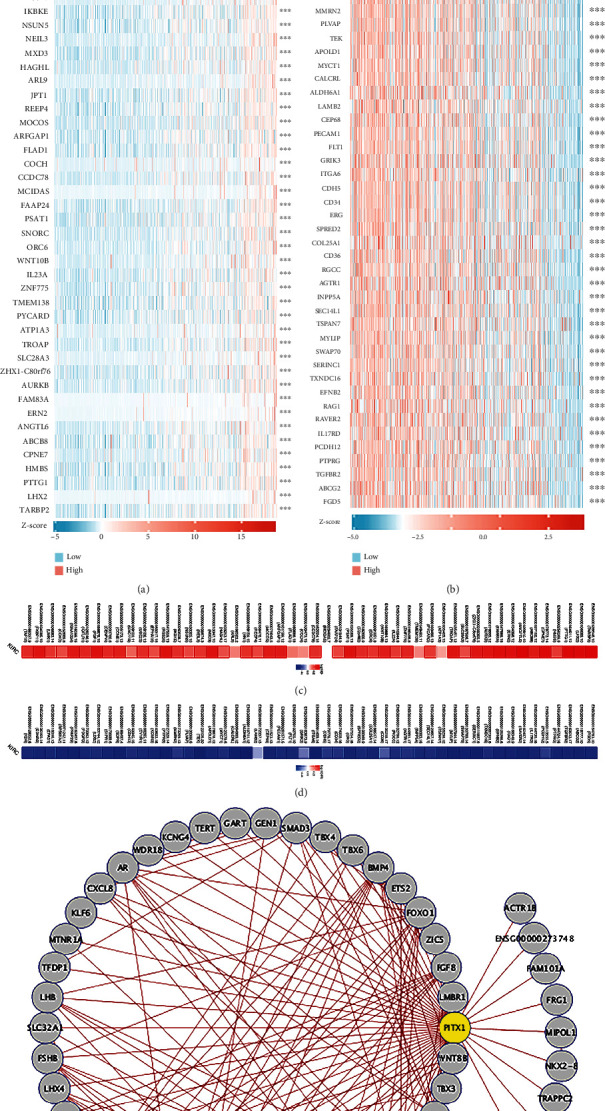
PITX1 coexpression genes in KIRC. (a, b) Heat maps showed the top 50 genes positively and negatively related to PITX1 by Spearman Correlation Coefficient in TCGA-KIRC cohort in KIRC, respectively. Red represents positively linked genes and blue represents negatively linked genes. (c, d) Survival heat maps of the top 50 genes positively and negatively related with PITX1 in KIRC. The survival heat maps showed the hazard ratios in the logarithmic scale (log_10_) for different genes. The red and blue modules meant higher and lower risks, respectively. The rectangles with borders implied the remarkable disadvantageous and advantageous results in prognostic analyses (*p* < 0.05). (e) A PPI network of PITX1 from the STRING database.

To explore the interactive network of PITX1, we checked the coexpression pattern of PITX1 mRNA in the TCGA-KIRC dataset and constructed a PPI network around PITX1. Two heat maps of the top 50 genes positively and negatively associated with PITX1 are shown in Figures 6(a) and 6(b), respectively. LIMK1and CCNO displayed strong positive correlations with PITX1. Notably, the top 50 positive genes had a high probability of becoming high-risk markers in KIRC, of which 44 genes had a high HR (*p* < 0.05) (Figure 6(c)). Nevertheless, 49 of the 50 negatively correlated genes had a low HR (*p* < 0.05) (Figure 6(d)). The PPI network was obtained with 51 nodes and 198 edges (Figure 6(e)). PITX1 is closely connected with some vital genes, such as FOXO1, EGR1, AR, SMAD3, RASAL1, and the TBX family.

### 3.6. PITX1-Related Signaling Pathways

**Figure 7:**
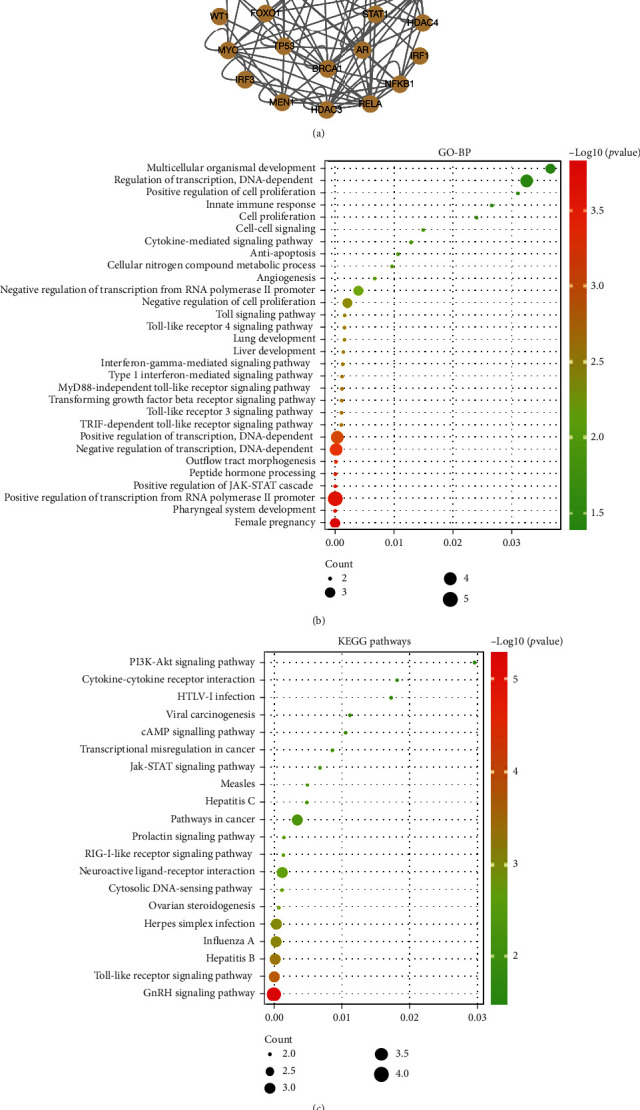
Enrichment analysis: (a) a PPI network among cooperative TFs of PITX1 from the TRRUST database; (b) GO-BP analysis; (c) KEGG pathway analysis. PPI: protein-protein interaction; GO-BP: Gene Ontology biological process; KEGG: Kyoto Encyclopedia of Genes and Genomes.

We use the TRRUST database to predict target genes of PITX1 downstream and other TFs that share targets with PITX1, and the results are listed in Figure 7(a), Supplementary Table 2, and Supplementary Table 3. GO-BP annotation showed that PITX1 mainly participated in positive/negative regulation of transcription from the RNA polymerase II promoter/DNA-dependent transforming growth factor beta receptor signaling pathway and positive regulation of JAK-STAT cascade (Figure 7(b)). KEGG pathways principally indicated enrichment in the GnRH signaling pathway, Toll-like receptor signaling pathway, pathways in cancer, cell proliferation, and transcriptional misregulation in cancer (Figure 7(c)), giving a clue of the underlying mechanism in the pathogenesis of KIRC.

## 4. Discussion

The differential expression of PITX1 mRNA has been observed in many types of cancer. Previous research has reported that PITX1 expression is decreased in numerous malignant tumors, which may be attributed to enrichment of binding gene promoters and regulation gene expression as a transcription factor. For instance, in prostate cancer and bladder cancer [15], PITX1 is expressed at low levels and suppresses tumorigenicity by downregulating the RAS pathway through the transcription target RASAL1. In other words, PITX has a close relationship with RASAL1, which is consistent with our outcome in the PPI network. In gastric cancer [16], decreased expression of PITX1 can predict shorter overall survival; it is also known that PITX1 binds to the apoptosis-related target gene PDCD5 to suppress GC cell proliferation. In malignant melanoma [17, 18], PITX1 directly binds to the hTERT promoter, retrains hTERT transcription, and eventually triggers the inhibition of telomerase activity and proliferation. In esophageal squamous cell carcinoma (ESCC) [14], PITX1 is expressed at low levels in tumors and is silenced by DNA hypermethylation. Hypermethylation of PITX1 is correlated with poor overall survival in ESCC. In breast cancer, PITX1 expression is enhanced, and high expression of PITX1 is associated with unfavorable clinical parameters and poor prognosis [19].

In our study, high-throughput RNA-seq data offered reliable evidence that PITX1 mRNA levels were elevated in KIRC tissue samples, and the wet experimental verification by qRT-PCR made our results more persuasive. Logistic regression analysis showed that high PITX1 mRNA expression was positively associated with advanced T and M stages, histologic grade, and pathologic stage. The ability to predict the survival outcomes of cancer patients is a crucial objective in cancer research. Our analysis revealed that patients with a high PITX1 mRNA expression were significantly associated with poor OS, DFS, and DSS. In addition, overall survival analysis based on the PITX1 expression level in different stratification subgroups similarly showed that upregulated PITX1 was associated with worse prognosis, except in the histologic grade G1/G2 group. Univariate and multivariate analyses of clinical data in the TCGA-KIRC dataset revealed age, M stage, and the PITX1 expression to be three independent prognostic risk factors. The higher AUCs for 1, 2, and 3 years of time-independent ROC curves further confirmed that the PITX1 signature has high sensitivity and specificity and can act as a reliable predictor of OS and DSS in KIRC patients. Our nomogram consisted of independent prognostic factors (age, M stage, and PITX1 mRNA expression), with a c-index of 0.739 (95% CI: 0.720-0.758), suggesting that the predicted survival probabilities were highly close to the actual proportions of incidence; therefore, the prediction model had a favorable accuracy. We believe that the model can directly contribute to clinicians predicting the individual patient′s death risk and guide patient assessment and therapeutic decision-making. In summary, our evidence emphasizes the important role of PITX1 mRNA in predicting the overall survival of patients with KIRC.

We also explored the interactive networks and functional clustering of the PITX1 gene by coexpression heat maps, PPIs, and enrichment analysis. For example, CCNO and LIMK1 were considered to be the closely coexpressed genes positively related to PITX1. Li et al. [20] showed that CCNO, a novel cyclin-like protein, was highly expressed in gastric cancer, and the proliferative properties of GC cells were strikingly inhibited by its knockdown. Inactivation of MAPK and Wnt signaling pathways may be involved in the suppressive effect of CCNO silencing. LIMK1 is a central regulator of cytoskeletal dynamics, LIM kinases promote the proliferation and survival of tumor cells, and LIM kinase inhibitors can affect microtubule organization and mitosis of tumor cells [21]. Therefore, our analysis showed that coexpression with PITX1 is credible. Meanwhile, we also observed that not only PITX1 has a significant impact on the prognosis of KIRC patients; most genes coexpressed with PITX1 in KIRC, whether positively or negatively related, also have a clear association with patient prognosis. This finding may provide a potential direction for identifying prognostic-related biomarkers of KIRC in the future. Although the function of PITX1 in KIRC is not clear, our analyses preliminarily revealed the interactive relationship between PITX1 and various genes, which provides a theoretical foundation for us to further explore the molecular mechanism of PITX1 in KIRC.

However, our work has certain limitations. Firstly, the gene expression analysis was based on the evaluation of the excised ex vivo tumor tissue samples, which represents only a “static” representation of the tumor biology, and the excision itself may impact on the biological processes of tumor cells. And an increasing portion of KIRC patients were treated with partial nephrectomy from which the tumor suffered an ischemia process, which may change the gene expression type and affect the accuracy of representing tumor biology [22]. Gene expression based on circulating tumor cells collected from patients' blood may reflect a real situation and “dynamic” process of the tumors and was reported as a valuable factor for the long-term prognosis [23]. And the integrated PITX1 expression analysis in tissues and liquid biopsy together may bring us a better view on understanding its negatively correlating with prognosis. Secondly, parameters in our prediction model are limited, for the TCGA database is short of detailed information, like basic diseases, medication, and surgical therapy, which may affect the patient's prognosis. Clinical, surgical, and imaging features associated with a poor prognosis have been extensively studied. The role of the functional imaging, circulating tumor cells, and various surgical approaches was also used for prognosis evaluation and showed great ability in predicting patient prognosis [24–26]. A possible integration with these prognostic markers in future prospective clinical studies may help us build a better prediction model which could potentially have a higher clinical impact for patients' management. Despite its limitations, this still was the first detailed report which examined associations between PITX1 mRNA expression with clinicopathological factors and prognosis in KIRC.

## 5. Conclusions

To sum up, our outcomes have provided a deeper insight that PITX1 mRNA was overexpressed in KIRC and high PITX1 mRNA expression is positively linked to advanced clinicopathological parameters and adverse prognosis in patients with KIRC. PITX1 mRNA has a significant prognostic value in patients with KIRC. The prediction model for overall survival may provide important references for clinical therapy and patient management. However, more efforts should focus on determining the potential biological function of PITX1 in vitro and in vivo.

## Figures and Tables

**Table 1:** A baseline data for KIRC patients on TCGA.

Clinical characteristics	Overall (n%)
Age	539
≤60	269 (49.9%)
>60	270 (50.1%)
Gender	539
Female	186 (34.5%)
Male	353 (65.5%)
Race	532
Asian	8 (1.5%)
Black or African American	57 (10.7%)
White	467 (87.8%)
Laterality	538
Left	252 (46.8%)
Right	286 (53.2%)
T stage	539
T1	278 (51.6%)
T2	71 (13.2%)
T3	179 (33.2%)
T4	11 (2%)
N stage	257
N0	241 (93.8%)
N1	16 (6.2%)
M stage	506
M0	428 (84.6%)
M1	78 (15.4%)
Pathologic stage	536
Stage I	272 (50.7%)
Stage II	59 (11%)
Stage III	123 (22.9%)
Stage IV	82 (15.3%)
Histologic grade	531
G1	14 (2.6%)
G2	235 (44.3%)
G3	207 (39%)
G4	75 (14.1%)
Primary therapy outcome	147
PD	11 (7.5%)
SD	6 (4.1%)
PR	2 (1.4%)
CR	128 (87.1%)
OS event	539
Alive	366 (67.9%)
Dead	173 (32.1%)
DSS event	528
Alive	420 (79.5%)
Dead	108 (20.5%)
PFI event	539
Alive	378 (70.1%)
Dead	161 (29.9%)

TCGA: The Cancer Genome Atlas; KIRC: kidney renal clear cell carcinoma; PD: progressive disease; SD: stable disease; PR: partial response; CR: complete response; OS: overall survival; DSS: disease-specific survival; PFI: progress-free interval.

**Table 2:** Relationship between PITX1 mRNA expression and clinical characteristics by logistic regression.

Characteristics	Total (*N*)	Odds ratio (OR)	*p* value
Age (>60 vs. ≤60)	539	1.259 (0.898-1.767)	0.182
Gender (male vs. female)	539	0.826 (0.578-1.178)	0.291
Race (white vs. black or African American)	532	0.795 (0.469-1.337)	0.389
Laterality (right vs. left)	538	0.719 (0.512-1.010)	0.058
T stage (T3+T4 vs. T1+T2)	539	2.354 (1.640-3.398)	<0.001
N stage (N1 vs. N0)	257	2.831 (0.956-10.349)	0.079
M stage (M1 vs. M0)	506	2.939 (1.764-5.039)	<0.001
Pathologic stage (stage III+IV vs. stage I+II)	536	2.775 (1.938-3.999)	<0.001
Histologic grade (G3+G4 vs. G1+G2)	531	2.384 (1.685-3.388)	<0.001
Primary therapy outcome (CR+PR vs. PD+SD)	147	1.115 (0.403-3.243)	0.835

Statistical significance *p* < 0.05.

**Table 3:** Univariate and multivariate Cox regression analyses of clinical characteristics associated with overall survival.

Characteristics	Total (*N*)	Univariate analysis		Multivariate analysis	
		Hazard ratio (95% CI)	*p* value	Hazard ratio (95% CI)	*p* value
Gender (female vs. male)	539	1.075 (0.788-1.465)	0.648		
Age (≤60 vs. >60)	539	0.567 (0.417-0.770)	<0.001	0.565 (0.369-0.866)	0.009
Race (black or African American vs. white)	524	0.856 (0.464-1.579)	0.618		
T stage (T3+T4 vs. T1+T2)	539	3.228 (2.382-4.374)	<0.001	1.695 (0.742-3.871)	0.211
N stage (N1 vs. N0)	257	3.453 (1.832-6.508)	<0.001	1.425 (0.701-2.896)	0.328
M stage (M1 vs. M0)	506	4.389 (3.212-5.999)	<0.001	2.949 (1.721-5.053)	<0.001
Pathologic stage (stage III+IV vs. stage I+II)	536	3.946 (2.872-5.423)	<0.001	1.081 (0.423-2.761)	0.871
Histologic grade (G3+G4 vs. G1+G2)	531	2.702 (1.918-3.807)	<0.001	1.638 (0.993-2.700)	0.053
PITX1 (high vs. low)	539	2.713 (1.959-3.757)	<0.001	1.998 (1.261-3.165)	0.003

Statistical significance *p* < 0.05. CI: confidence interval.

## Data Availability

The data included in the current study are available in the TCGA database (https://cancergenome.nih.gov/) and GEO database (https://www.ncbi.nlm.nih.gov/). Original experimental data are available if required.

## Supplementary Materials

Supplementary 1 Supplementary Table 1: the basic information of 10 patients with KIRC.

Supplementary 2 Supplementary Table 2: transcription factor PITX1 and its target genes.

Supplementary 3 Supplementary Table 3: other transcription factors that share targets with PITX1.

Supplementary 4 Supplementary Figure 1: the imaging data of 10 patients with KIRC.
