# Supplementary material for: Upregulated Transcription Factor PITX1 Predicts Poor Prognosis in Kidney Renal Clear Cell Carcinoma-Based Bioinformatic Analysis and Experimental Verification
Source: Dis Markers. 2021 Nov 23;2021:7694239. doi: 10.1155/2021/7694239 (PMC8633854; doi:10.1155/2021/7694239)
Supplement: Supplementary 1 — Supplementary Table 1: the basic information of 10 patients with KIRC. [file 7694239.f1.docx]

Supplementary table 1: The basic information of 10 patients with KIRC.

| **Number** | **Name** | **Gender** | **Age** | **Laterality** | **TNM stage** | **Surgical procedure** |
| --- | --- | --- | --- | --- | --- | --- |
| a | L. Tian | female | 60 | right | T1N0M0 | Robot-assisted radical resection |
| b | B. X. Feng | male | 61 | right | T1N0M0 | Laparoscopic radical resection |
| c | S. S. Mi | male | 75 | left | T1N0M0 | Laparoscopic radical resection |
| d | S. Y. Tai | male | 66 | right | T1N0M0 | Laparoscopic radical resection |
| e | Z. H. Gu | female | 75 | right | T3N0M0 | Robot-assisted radical resection |
| f | S. H. Zhang | female | 67 | right | T1N0M0 | Robotic-assisted partial nephrectomy |
| g | J. J. Wang | male | 79 | right | T1N0M0 | Robot-assisted radical resection |
| h | Q. S. Wu | male | 58 | left | T1N0M0 | Robot-assisted radical resection |
| i | B. X. Zhao | male | 74 | left | T1N0M0 | Robot-assisted radical resection |
| j | Z. X. Wang | female | 72 | right | T1N0M0 | Robotic-assisted partial nephrectomy |
