# Supplementary material for: Upregulated Transcription Factor PITX1 Predicts Poor Prognosis in Kidney Renal Clear Cell Carcinoma-Based Bioinformatic Analysis and Experimental Verification
Source: Dis Markers. 2021 Nov 23;2021:7694239. doi: 10.1155/2021/7694239 (PMC8633854; doi:10.1155/2021/7694239)
Supplement: Supplementary 2 — Supplementary Table 2: transcription factor PITX1 and its target genes. [file 7694239.f2.docx]

Supplementary table 2: Transcription factor PITX1 and its target genes.

| **TF** |  | **Target** | **Mode of regulation** |
| --- | --- | --- | --- |
| PITX1 | | DUX4 | Unknow |
| PITX1 | | FSHB | Unknow |
| PITX1 | | GNRH1 | Repression |
| PITX1 | | HES1 | Repression |
| PITX1 | | IFNA1 | Repression |
| PITX1 | | IRF2 | Repression |
| PITX1 | | IRF7 | Repression |
| PITX1 | | JUN | Repression |
| PITX1 | | LHB | Unknow |
| PITX1 | | NKX3-1 | Repression |
| PITX1 | | PRL | Activation |
| PITX1 | | RUNX1 | Repression |
| PITX1 | | RUNX2 | Repression |
| PITX1 | | TBX4 | Unknow |
| PITX1 | | TERT | Repression |
| PITX1 | | TRIB2 | Repression |
| TF: transcription factors | | |  |
