# Supplementary material for: Upregulated Transcription Factor PITX1 Predicts Poor Prognosis in Kidney Renal Clear Cell Carcinoma-Based Bioinformatic Analysis and Experimental Verification
Source: Dis Markers. 2021 Nov 23;2021:7694239. doi: 10.1155/2021/7694239 (PMC8633854; doi:10.1155/2021/7694239)
Supplement: Supplementary 3 — Supplementary Table 3: other transcription factors that share targets with PITX1. [file 7694239.f3.docx]

Supplementary table 3: Other transcription factors that share targets with PITX1.

| **TF** | ***P* value** | **FDR** |
| --- | --- | --- |
| PITX2 | 1.10E-07 | 6.28E-07 |
| FOXO1 | 1.29E-06 | 5.18E-06 |
| SMAD3 | 2.45E-06 | 8.85E-06 |
| TNFAIP3 | 6.73E-06 | 2.03E-05 |
| ESR1 | 3.52E-05 | 7.83E-05 |
| MEN1 | 3.69E-05 | 8.14E-05 |
| ETS1 | 6.20E-05 | 1.22E-04 |
| IRF3 | 8.04E-05 | 1.52E-04 |
| AR | 8.17E-05 | 1.54E-04 |
| RELA | 1.16E-04 | 2.05E-04 |
| NFKB1 | 1.17E-04 | 2.06E-04 |
| ESR2 | 1.27E-04 | 2.21E-04 |
| HDAC4 | 1.84E-04 | 3.00E-04 |
| HDAC3 | 2.17E-04 | 3.44E-04 |
| JUN | 2.82E-04 | 4.28E-04 |
| RUNX3 | 2.89E-04 | 4.38E-04 |
| VDR | 7.75E-04 | 9.98E-04 |
| IRF1 | 8.72E-04 | 1.10E-03 |
| WT1 | 1.05E-03 | 1.29E-03 |
| BRCA1 | 1.12E-03 | 1.37E-03 |
| CREB1 | 2.64E-03 | 2.96E-03 |
| STAT1 | 2.76E-03 | 3.07E-03 |
| MYC | 3.77E-03 | 4.08E-03 |
| TP53 | 8.52E-03 | 8.81E-03 |
| Statistical significance *p*<0.05; TF: transcription factors; FDR: false discovery rate | | |
