# Supplementary figures and images for: Upregulated Transcription Factor PITX1 Predicts Poor Prognosis in Kidney Renal Clear Cell Carcinoma-Based Bioinformatic Analysis and Experimental Verification
Source: Dis Markers. 2021 Nov 23;2021:7694239. doi: 10.1155/2021/7694239 (PMC8633854; doi:10.1155/2021/7694239)

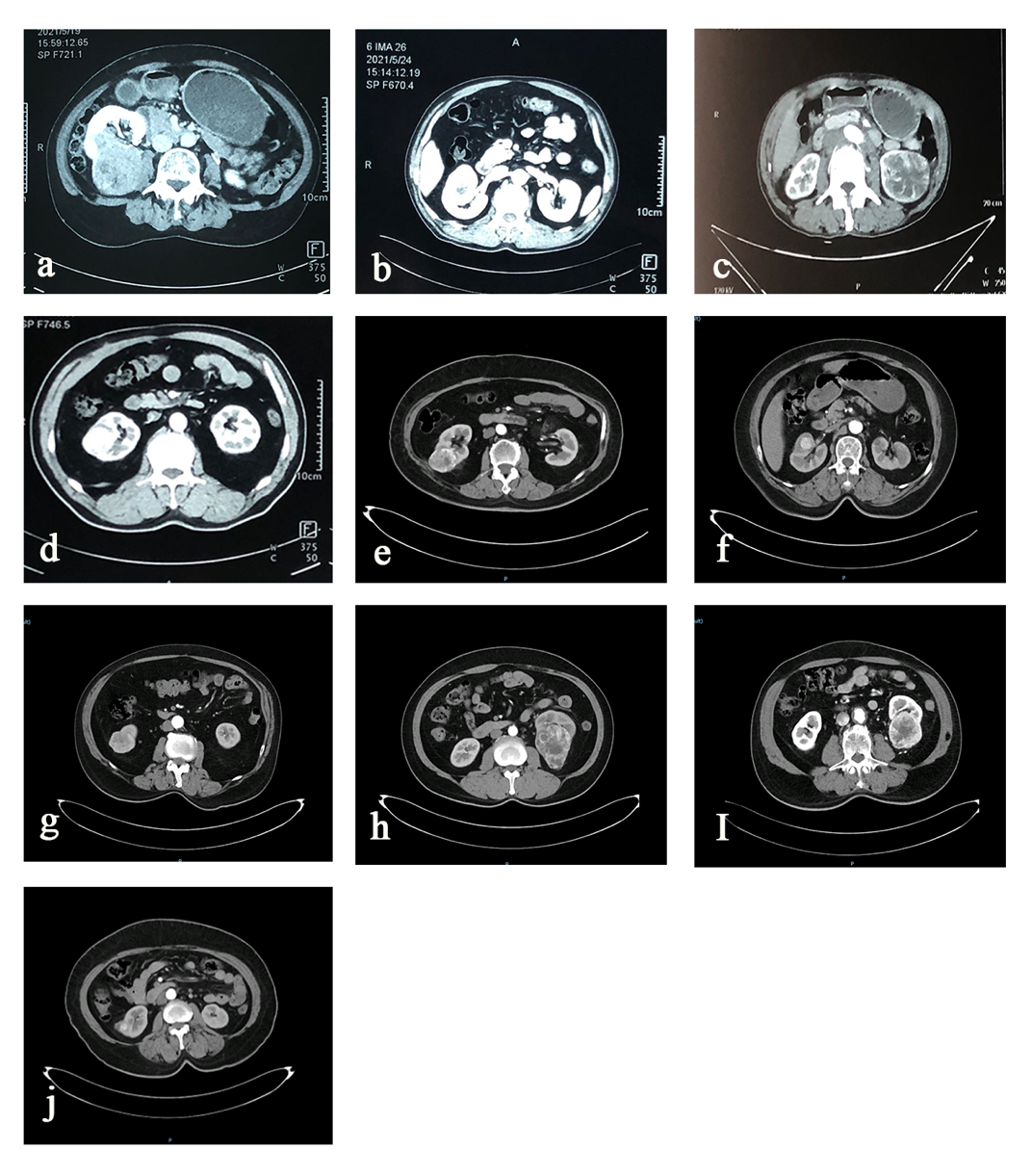
Figure S1. The imaging data of 10 patients with KIRC.

Supplement: Supplementary 4 — Supplementary Figure 1: the imaging data of 10 patients with KIRC. [file 7694239.f4.docx]
